# Temporal serum neurofilament light chain concentrations in sheep inoculated with the agent of classical scrapie

**DOI:** 10.1371/journal.pone.0299038

**Published:** 2024-02-23

**Authors:** Quazetta Brown, Eric Nicholson, Chong Wang, Justin Greenlee, Hannah Seger, Susan Veneziano, Eric Cassmann

**Affiliations:** 1 United States Department of Agriculture, Virus and Prion Research Unit, National Animal Disease Center, Agricultural Research Service, Ames, Iowa, United States of America; 2 Oak Ridge Institute for Science and Education, Oak Ridge, Tennessee, United States of America; 3 Department of Biomedical Sciences, Iowa State University College of Veterinary Medicine, Christensen, Ames, United States of America; 4 Department of Veterinary Diagnostic and Production Animal Medicine, College of Veterinary Medicine, Iowa State University, Ames, Iowa, United States of America; National Institute of Allergy and Infectious Diseases, UNITED STATES

## Abstract

**Objective:**

Neurofilament light chain (Nf-L) has been used to detect neuroaxonal damage in the brain caused by physical injury or disease. The purpose of this study was to determine if serum Nf-L could be used as a biomarker for pre-symptomatic detection of scrapie in sheep.

**Methods:**

Four sheep with prion protein genotype AVQQ were intranasally inoculated with the classical scrapie strain x124. Blood was collected every 4 weeks until 44 weeks post-inoculation, at which point weekly collection commenced. Serum was analyzed using single molecule array (Quanterix SR-X) to evaluate Nf-L concentrations.

**Results:**

Scrapie was confirmed in each sheep by testing homogenized brainstem at the level of the obex with a commercially available enzyme immunoassay. Increased serum Nf-L concentrations were identified above the determined cutoff during the last tenth of the respective incubation period for each sheep. Throughout the time course study, PrP^Sc^ accumulation was not detected antemortem by immunohistochemistry in rectal tissue at any timepoint for any sheep. RT-QuIC results were inconsistently positive throughout the timepoints tested for each sheep; however, each sheep had at least one timepoint detected positive. When assessing serum Nf-L utility using receiver operator characteristic curves against different clinical parameters, such as asymptomatic and symptomatic (pruritus or neurologic signs), results showed that Nf-L was most useful at being an indicator of disease only late in disease progression when neurologic signs were present.

**Conclusion:**

Serum Nf-L concentrations in the cohort of sheep increased as disease progressed; however, serum Nf-L did not increase during the presymptomatic window. The levels increased substantially throughout the final 10% of the animals’ scrapie incubation period when other clinical signs were present. Serum Nf-L is not a reliable biomarker for pre-clinical detection of scrapie.

## Introduction

Biological markers, or biomarkers, are substances produced in the body that can be measured and used to monitor disease progression and onset [1]. Biomarkers can be classified as specific and non-specific, where non-specific markers are produced by several pathologic processes and are subsequently not conclusive for a single disease. Neurofilament light (Nf-L) is a structural protein of neurons and a non-specific biomarker [2] of neurodegenerative diseases. Nf-L levels increase in the serum and cerebrospinal fluid (CSF) after neuroaxonal damage [3–6].

Transmissible spongiform encephalopathies (TSEs), commonly referred to as prion diseases, are fatal neurodegenerative diseases that affect humans and animals [7]. Prion diseases are caused by the misfolding of normal cellular prion protein (PrP^C^) and accumulation of the disease associated form (PrP^Sc^) [7]. Well known prion diseases are chronic wasting disease (CWD) in cervids, bovine spongiform encephalopathy (BSE) in bovids, Creutzfeldt-Jakob disease (CJD) in humans, and scrapie in goats and sheep.

Numerous studies have investigated the utility of serum Nf-L concentration as a diagnostic and prognostic tool for human neurodegenerative diseases [8–29]. In human CJD, CSF Nf-L is elevated before the RT-QuIC amplification assay can detect CSF prion seeding activity, and serum Nf-L is increased before clinical disease onset [27]. Patients with fast incubating inherited prion disease demonstrate increased Nf-L around 2 years prior to onset of clinical signs [30, 31]; in contrast, patients with slowly progressing inherited prion diseases had serum Nf-L spikes with a narrow pre-symptomatic window [31]. In a study of sheep with naturally occurring field scrapie, serum Nf-L was elevated in sheep. Elevations of Nf-L were present in scrapie positive sheep with and without clinical neurologic signs–defined as proprioceptive deficits and a low body condition score [32].

Current antemortem diagnosis of classical scrapie in sheep involves obtaining biopsies of rectal mucosal, third eyelids, or tonsils to examine lymphoid tissue for the presence of PrP^Sc^ [33–36]; however, these tests have limitations and require skilled personnel to perform [34]. Drawing blood is a less invasive method and is simpler than the current methods for antemortem testing of PrP^Sc^. The facile nature of blood collection would allow easy screening of animals to select subgroups of large herds for more rigorous and definitive testing. The purpose of this study was to compare serum Nf-L concentration with disease progression of sheep inoculated with a fast-incubating strain of classical scrapie and to determine the earliest point of serum Nf-L elevation.

## Materials and methods

### Ethics statement

All experiments were conducted in inspected and approved facilities consistent with the requirements for import and use of prion agents by the US Department of Agriculture, Animal and Plant Health Inspection Service, Veterinary Services. The studies were done in accordance with the Guide for the Care and Use of Laboratory Animals (Institute of Laboratory Animal Resources, National Academy of Sciences, Washington, DC, USA) and the Guide for the Care and Use of Agricultural Animals in Research and Teaching (Federation of Animal Science Societies, Champaign, IL, USA). The protocols were approved by the Institutional Animal Care and Use Committee at the National Animal Disease Center (protocol number: ARS 2020–910), which requires species-specific training in animal care for all staff handling animals.

### Animal use

Sheep that were used for the experiment were sourced from a scrapie-free flock housed at the USDA National Animal Disease Center (NADC) in Ames, IA. All sheep were Suffolk males with the same prion protein genotype: VRQ/ARQ.

### Inoculum preparation and inoculation

The inoculum was sourced from the brainstem at the level of the obex from an VRQ/ARQ prion protein genotype sheep that was inoculated with the x124 scrapie strain [37]. The brain homogenate was prepared by homogenization in phosphate buffered saline (PBS) to a 10% w/v solution with a BeadMill 24 homogenizer (Fisher Scientific Co., Pittsburgh, PA, USA). The sheep were intranasally inoculated with 1 mL of a 10% w/v (0.1 grams) homogenate when they were approximately 3 months old. The sheep were assessed daily for the presence of clinical signs of scrapie, including wool loss, pruritus (observed as wool loss combined with rubbing or biting at wool), ataxia, and proprioceptive deficits. When unequivocal neurologic symptoms were observed, the animal was euthanized and necropsied. Animals were not allowed to develop severe, end-stage disease. Animals were euthanized via intravenous administration of sodium pentobarbital according to label directions or as directed by a veterinarian.

### Sample collection

Every 4 weeks, whole blood and rectal mucosal biopsies were collected from each sheep. At 44 weeks post-inoculation, blood samples were taken weekly. A 3–4 mm diameter piece of rectal biopsy tissue was dissected and placed in 1.5 mL microcentrifuge tubes immediately after each biopsy and stored at -80°C for RT-QuIC assay. The remaining rectal tissues were fixed in 10% buffered formalin for 36 hours then switched to 70% ethanol. Tissues were embedded in paraffin wax to be evaluated for the presence of PrP^Sc^ by immunohistochemistry. After collection, blood was centrifuged at 22,000 rpm for 25 minutes whereafter serum was collected, aliquoted, and stored at -80°C. Tissues were collected at necropsy including brain, eye (with optic nerve), pituitary gland, trigeminal ganglia, spinal cord, sciatic nerve, rectal mucosal junction, bladder, muscles (triceps heart, biceps femoris, diaphragm, psoas major, masseter, eye), thyroid, esophagus, thymus, tongue, lung, pancreas, adrenal gland, liver, kidney, spleen, haired skin, rumen, reticulum, omasum, abomasum, ileal-cecal junction, jejunum, trachea, turbinate, nose skin, parotid salivary gland, palatine tonsil, nasal pharyngeal tonsil, 3rd eyelid, and lymph nodes (prescapular, retropharyngeal, popliteal, mesenteric). We collected both bilateral tissues for palatine tonsils and retropharyngeal lymph nodes. Each bilateral tissue was assigned a number, 1 or 2, for differentiation purposes.

For evaluating serum Nf-L concentrations in healthy sheep, whole blood was collected from thirty sheep in the NADC scrapie free flock.

### Enzyme immunoassay

Frozen samples were thawed and homogenized in PBS to a 20% (w/v) concentration. The presence of PrP^Sc^ in the rectal mucosa and brain stem, at the level of the obex was confirmed by the commercial enzyme immunoassay kit (HerdChek; IDEXX Laboratories, Westbrook, ME) according to kit instructions. The negative cutoff was determined by measuring the mean optical density of the negative controls +0.180.

### Immunohistochemistry

Fixed tissues from necropsy were processed routinely and stained with hematoxylin and eosin; brain tissues were assessed for the presence of spongiform change. For rectal mucosal biopsies, three progressive sections were obtained at different depths in the paraffin block to assess varying numbers of lymphoid follicles. The depth of sections corresponded to approximately 50, 100, and 150 microns deep.

The slides were incubated at 55–60°C for 60 minutes. The slides were deparaffinized and hydrated in a fume hood through graded alcohols. Following a rinse in water, the slides were exposed to 95–100% formic acid for 5 minutes. Following the formic acid, slides were put in a 1x reaction buffer bath (Ventana, Roche) for 2 minutes, and this was repeated three times, adding new reaction buffer each time. Slides were then rinsed with RO water for 1 minute then moved 1x DIVA Decloaker (BioCare Medical, Pacheco, California). Slides were placed in a decloaking chamber (DC2002 Decloaking Chamber; BioCare Medical, Pacheco, California) and heated to temperatures of 121°C and 95°C for 20 minutes and 25 minutes, respectfully. Once slides finished the cycle in the Decloaker, they were placed in a 1:1 bath of RO water and DIVA(1x) for 2 minutes. The slides were rinsed with RO water for 2 minutes, and this was repeated twice. After the rinses, the slides were placed on an automated staining machine (Ventana Discovery XT; Roche, Tucson, Arizona) where an anti-PrP^Sc^ antibody (F99) was applied to all the slides at a concentration of 8 μg/mL for 44 minutes to detect the presence of PrP^Sc^. Known positive and negative controls for classical scrapie in sheep tissue (lymph node and brain) were included in each run.

### Real-time quaking-induced conversion

#### Rectal biopsy homogenization

Rectal biopsy samples were weighed and placed in homogenate tubes containing 1.0 mm zirconia/silica beads. Phosphate Buffered Saline (1X PBS) was added to create 10% (w/v) homogenate (i.e., 0.1 g tissue plus 1 mL 1X PBS) and a ¼ inch ceramic bead was added to each tube. Samples were homogenized in a BeadMill tissue homogenizer (Fisher) at Power 6, 1 min on, 5-minute rest with 3 replicates at 4°C. The resulting homogenate was stored at -80°C until used to seed RT-QuIC reactions.

#### Real-time quaking induced conversion

The RT-QuIC reaction mixture was composed of 10 mM phosphate buffer, pH 7.0, 400 mM NaCl, 0.1 mg/mL recombinant bank vole prion protein (M109) [38, 39], 10 μM thioflavin T (ThT), and 1 mM EDTA tetrasodium salt. Aliquots of reaction mixture (98 μL) were loaded into black walled, clear optic-bottomed 96-well plates (Nunc, Thermo Fisher Scientific, USA). Reactions were seeded with 2 μL of rectal biopsy homogenate diluted 10^−2^ in 1X PBS with 0.05% sodium dodecyl sulfate. Appropriate positive and negative control seeds were included on each plate– 2 μL genotype matched scrapie positive sheep brain homogenate and uninoculated sheep brain homogenate prepared as a 10% (w/v) homogenate and further diluted 10^−2^ using the same reaction mixture. Rectal biopsy tissue from scrapie free sheep was prepared as described for the experimental samples and used as an additional negative control. Plates were sealed with plate film and incubated at 42°C in a BMG FLUOstar Omega plate reader with cycles of 1 minute shaking (700 rpm double orbital) and 1 minute rest for 100 h. ThT fluorescence was measured every 15 minutes (bottom read, excitation 448 nm, emission 482 nm, manual gain 1200 or 1400 (instrument dependent), 20 flashes per well, 0.2 second settling time) [38, 40, 41].

Samples and controls were run in quadruplicate and ThT fluorescence data was measured as the average of four replicates for each time point for each sample. To be considered positive at least 2 replicates out the four must be positive. The threshold for determining a positive sample was calculated as the mean value of ThT fluorescence for the first 3 hours of 1 negative control sample plus 10 standard deviations from the mean. Time to threshold (lag time) was calculated by BMG MARS analysis software and presented as the average of 4 replicates per sample [40–43].

#### Single molecule array

Nf-L levels in the sheep serum were measured using the Nf-light advantage kit assay on the SR-X Biomarker Detection system (Quanterix, Lexington, MA) according to kit instructions. Briefly, the sheep’s serum was thawed from -80°C. The samples were diluted 4x and ran as triplicates on each plate. Individual results were further analyzed only if those values had fitted concentration coefficient of variation less than 0.2. The average coefficient of variation in the final dataset was 0.1246.

## Results

### Confirmation of scrapie by enzyme immunoassay

To assess the presence of PrP^Sc^ in specific necropsy tissues, enzyme immunoassay was performed. PrP^Sc^ was detected in the brain (at the level of the obex) for all sheep. Lymphoid tissues that were tested included: retropharyngeal lymph node (RPLN), palatine tonsil, and postmortem rectal biopsies. Three out of four sheep had PrP^Sc^ detection in lymph tissue (Table 1). All sheep had positive enzyme immunoassay (EIA) results in the brainstem at the level of the obex. Retropharyngeal lymph nodes from sheep 2111 and 2113 were unilaterally positive by EIA; whereas, the RPLNs from 2112 and 2115 were bilaterally negative for PrP^Sc^. No PrP^Sc^ was detected in any sheep’s postmortem rectal biopsy (no sample was available to test sheep 2112). Detection of PrP^Sc^ in the palatine tonsil by EIA was unilateral in positive samples.

**Table 1:** Enzyme immunoassay results for brainstem and lymphoid tissue.

Sample	Sheep ID			
	2111	2112	2113	2115
Brain	+	+	+	+
RPLN	+	-	+	-
Rectal biopsy	-	ns	-	-
Palatine tonsil	+	-	+	+

RPLN, retropharyngeal lymph node; ns, no sample; “—” indicates PrP^Sc^ was not detected.

Results for selected necropsy tissues are displayed. Brain regions (obex, cerebrum, cerebellum, thalamus) were positive by EIA. Sheep 2111 and 2113 were unilaterally positive for PrP^Sc^ in the RPLN. No PrP^Sc^ was detected in the postmortem rectal biopsy samples by EIA. All detections of PrP^Sc^ in palatine tonsils of the three sheep indicated were unilaterally positive.

### Antemortem rectal biopsies

#### Detection of PrP^Sc^ by immunohistochemistry.

No antemortem rectal biopsy samples were positive for PrP^Sc^ by IHC. These tissues were defined as “non-detect” when at least six follicles were present, and IHC was negative. Samples with less than six follicles were classified as “insufficient follicles” when IHC was negative [44, 45].

#### Temporal characteristics of antemortem rectal biopsy

To investigate the characteristics of rectal biopsies over time, we counted the number of lymphoid follicles at three separate depths for each timepoint throughout the scrapie incubation period. Follicle presence was often more abundant deeper in the rectal biopsy tissue. However, the difference in the number of follicles per depth was not statistically significant (generalized linear model, α = .05). The number of total follicles for each sheep’s biopsy decreased over time (generalized linear model, p < .0001).

#### Detection of PrP^Sc^ by real-time quaking induced conversion.

**Fig 1:**
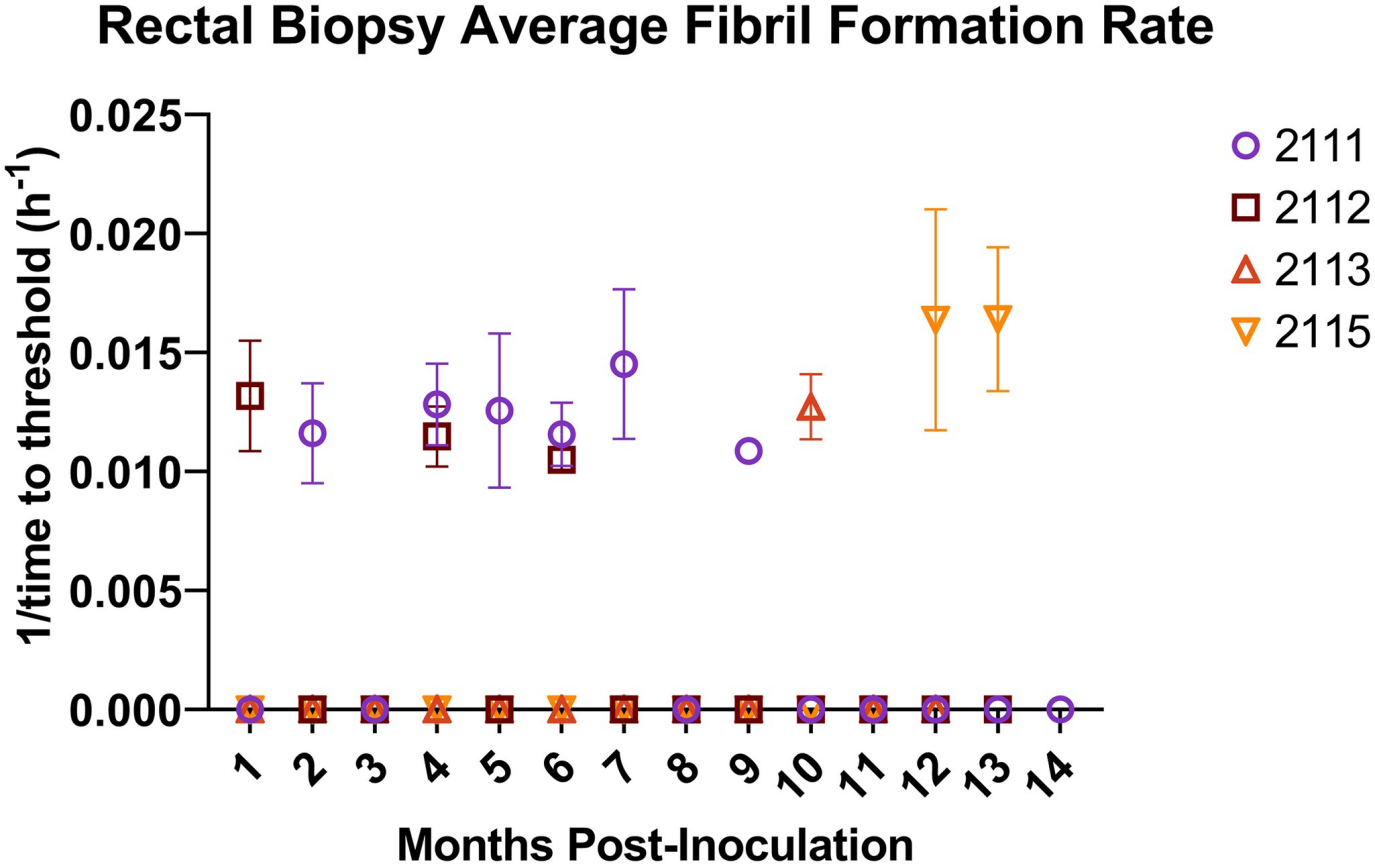
RT-QuIC detection of prion seeding activity in rectal biopsy tissue. Each color/shape combination represents a different animal that was used in the study. Every sheep sample per timepoint had 4 technical replicates performed. Samples were considered RT-QuIC positive when 2/4 replicates reached threshold–a data point is present when a sample was positive for that timepoint. Negative samples appear at the baseline of the x-axis. The error bars indicate standard deviation of technical replicates.

To assess the earliest timepoint at which prion seeding activity could be detected in rectal tissues, RT-QuIC was performed. Samples were run with 4 technical replicates; a sample was considered positive if amplification was detected in at least 2 technical replicates. All sheep had positive RT-QuIC at least once throughout the incubation period. The earliest detection of PrP^Sc^ was one month post inoculation (mpi). Fig 1 shows the frequency of RT-QuIC detections throughout the scrapie incubation period in rectal biopsy tissue. The highest number of detections in rectal tissue for a single sheep was 6/14; while the lowest number of positive detections was 1/11.

#### Measurement of serum Nf-L by single molecule array

To evaluate normal serum Nf-L concentrations in healthy sheep, we collected blood from thirty sheep in the NADC scrapie-free breeding flock and pre-inoculation serum from the four sheep in this experiment (n = 34). Healthy sheep were comprised of eleven rams, four wethers, and nineteen ewes with ages ranging from 10 weeks to 6.9 years-old (mean 2.6 years, median 1.9 years). The average serum Nf-L concentration was 4.72 pg/mL (3.986–5.460 pg/mL, 95% CI). There was no difference in serum Nf-L concentration between female and male sheep, 4.111 and 5.498 pg/mL, respectively (p = .0559). Younger sheep had higher Nf-L during the first several months of life. A Pearson correlation coefficient was computed to assess the relationship between age and serum Nf-L, r = -.575, p = .0004.

**Fig 2:**
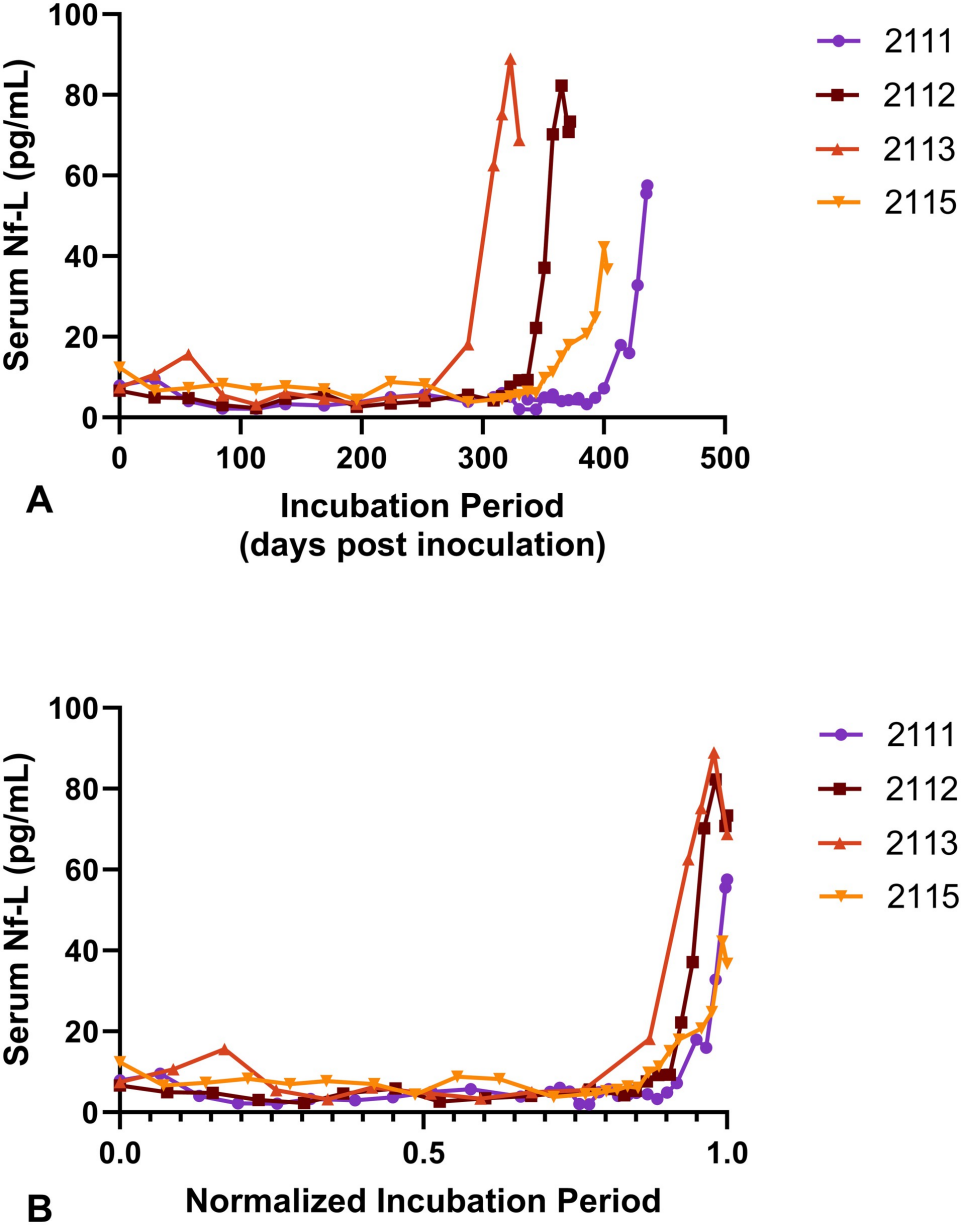
Serum neurofilament light (Nf-L) concentrations. (A) The incubation period for each animal was between 330 to 436 days post inoculation. Four lines are shown representing serum Nf-L concentrations for each animal used in the study. (B) To meaningfully compare changes in serum Nf-L concentrations between animals incubating scrapie, each sample timepoint was calculated as a fraction of that animal’s scrapie incubation period. Although, Nf-L elevations were detected at different chronological points between animals, elevations in serum Nf-L occurred at similar fractional times of the incubation period once that timeframe was normalized. All animals have a notable rise in Nf-L levels during the final 10% of the scrapie incubation period.

The four sheep inoculated with classical scrapie had an average serum Nf-L concentration of 5.43 pg/mL throughout the asymptomatic portion of the incubation period which included 44 total data points (minimum, 2.09 pg/mL; maximum, 15.66 pg/mL; median, 4.99 pg/mL; 95% CI 4.65–6.21 pg/mL). A single sheep had a transient serum Nf-L concentration of 15.66 pg/mL at 57 days post inoculation that subsequently decreased until the symptomatic period. in all four sheep, the serum Nf-L levels increased during the late stages of the incubation period concomitant with the appearance of clinical signs consistent with scrapie (Fig 2 and S1 Table).

#### Correlation of serum Nf-L with clinical signs

To compare the serum concentration of Nf-L with the appearance of clinical signs we considered wool loss and pruritus as early clinical signs. Neurologic signs were defined as ataxia and proprioceptive deficits, and these were late-stage clinical signs. Nf-L levels from a population of healthy sheep and pre-symptomatic experimental sheep were used as the control population. There was no difference in Nf-L serum concentrations between healthy control sheep and blood taken from asymptomatic experimental sheep (p = .1627). To determine the correlation of Nf-L with clinical and neurologic symptoms, we recorded symptoms throughout the incubation period of the sheep. All sheep progressed from asymptomatic to wool loss/pruritus to having neurologic symptoms. We found that elevated serum Nf-L concentrations were significantly correlated with neurologic signs (Pearson Partial Correlation, r = .767, p < 0.0001). However, increased Nf-L concentrations were not correlated with pruritis alone (Pearson Partial Correlation, r = -.161, p = 0.0785).

**Fig 3:**
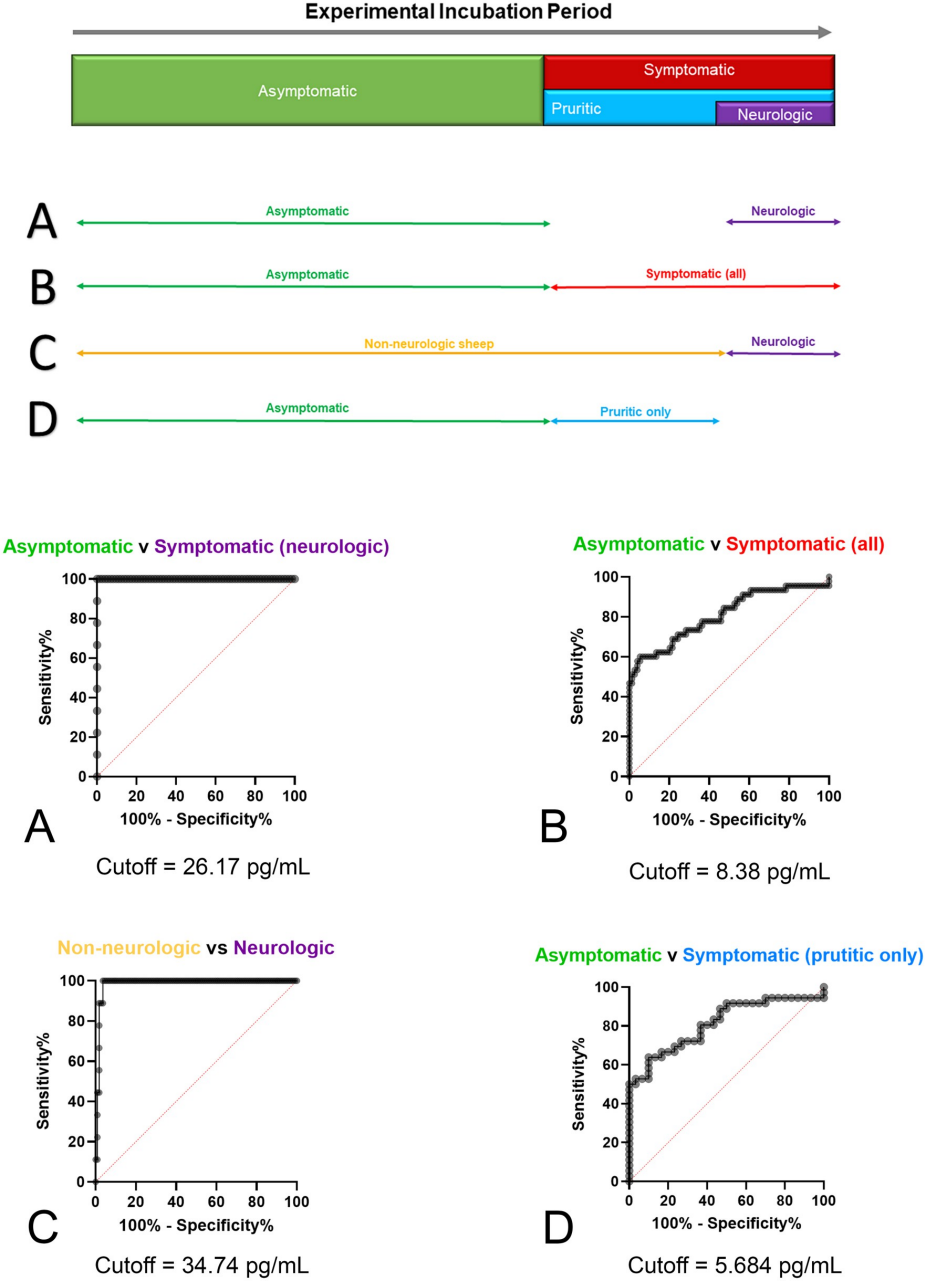
Determination of cutoff values for serum Nf-L that differentiated clinical stages of sheep scrapie using receiver operator characteristic (ROC) curves. The appearance of clinical signs is displayed in a timeline overlay of the incubation period (not to scale). Arrows to the right of A-D illustrate the appearance of clinical signs over time. The comparisons, A-D, correspond to the ROC curves below. (A) The serum Nf-L cut-off for asymptomatic sheep compared to sheep with neurologic signs. (B) The serum Nf-L cut-off for asymptomatic compared to all symptomatic sheep. (C) The serum Nf-L cut-off for non-neurologic sheep compared to sheep with neurologic signs. (D) The serum Nf-L cut-off for asymptomatic sheep compared to only the sheep with pruritus; prior to when those sheep developed neurologic signs.

Receiver operating characteristic (ROC) curves were used to determine diagnostic cutoffs of serum Nf-L for differentiating clinical stages of scrapie (Fig 3). Cutoff values were determined using Youden’s index (J). When comparing Nf-L levels of asymptomatic sheep vs sheep with neurologic symptoms the cutoff value was 26.17 pg/mL corresponding to 100% sensitivity (70.1–100%, 95% CI) and 100% specificity on the ROC curve (95.6–100%, 95% CI). The cutoff value for Nf-L when asymptomatic sheep and pruritic sheep were compared to sheep with neurologic signs (all non-neurologic vs neurologic) was 34.74 pg/mL. This value corresponded to 100% sensitivity (70.1–100%, 95% CI) and 96.4% specificity (91.0–98.6%, 95%CI). Timepoint serum Nf-L levels reached this cutoff during the final tenth of the scrapie incubation period (Table 2). A comparison between asymptomatic sheep and symptomatic sheep (all) yielded a Nf-L cutoff value of 8.38 pg/mL with 60% sensitivity (45.5–73%, 95% CI) and 94.6% specificity (86.9–97.9%, 95% CI) on the ROC curve. The cutoff value to detect pruritus alone (asymptomatic vs pruritus only) was 5.68 pg/mL with a sensitivity of 63.9% (47.6–77.5%, 95% CI) and specificity of 90% (74.4–96.5%, 95% CI).

**Table 2:** Non-neurologic vs neurologic.

Animal ID	Non-neurologic vs Neurologic			
	1^st^ Nf-L value above cutoff (pg/mL)	Days post-inoculation	Total incubation period (days)	Proportion of inoculation period
2111	55.5	435	436	0.99
2112	37.1	351	372	0.94
2113	62.5	309	330	0.94
2115	42.2	400	403	0.99

Below shows how Nf-L performed at differentiating non-neurologic sheep from sheep with neurologic clinical signs (ataxia and proprioceptive deficits). The data of non-neurologic sheep included non-inoculated control sheep and experimental sheep that were not expressing clinical signs. The cutoff value was 34.74 pg/mL. This value corresponds with a 100% sensitivity and 96.4% specificity on the ROC curve. This table includes the number of days post inoculation when the animal breached the threshold of the cutoff and the proportion of the incubation period when this happened.

## Discussion

Serum Nf-L levels stayed relatively low and consistent throughout the sheep’s incubation period. Only toward the end of the experiment did Nf-L levels begin to rise. Nf-L was a good indicator for neurologic disease. Levels above the determined cutoff of 34.74 pg/mL were able to differentiate non-neurologic sheep from sheep with neurologic signs (ataxia or proprioceptive deficits). However, this didn’t occur until the last tenth of the incubation period. Our cutoff of 34.74 pg/mL was similar to a previously reported cutoff of 31 pg/mL by Zetterberg et al [32]. In the paper by Zetterberg and colleagues, scrapie positive sheep without neurologic signs had serum Nf-L levels similar to our neurologic sheep, and the authors concluded that serum Nf-L increased prior to clinical neurologic disease. When we attempted to differentiate asymptomatic sheep from earlier clinical signs like pruritus/wool loss, the sensitivity and specificity declined substantially. We conclude that in our model, serum Nf-L was not a good pre-symptomatic biomarker for sheep with classical scrapie even when detectable levels of PrP^Sc^ are present in the host as determined by RT-QuIC. One possible explanation for this difference could be the speed of scrapie incubation relative to inherited human prion diseases. Mok et al, recently reported that humans with fast progressing inherited prion diseases had no pre-symptomatic window for detection of Nf-L; whereas, Nf-L increased 4 years prior to the onset of neurologic disease in patients with slowly progressing inherited prion disease [31]. Further compounding the discrepancy between scrapie incubation time and inherited human prion diseases, we used a fast-incubating scrapie strain; it’s possible that a slower incubating scrapie strain may have influenced the rate and concentration of Nf-L released in the blood.

The discrepancy between our study and Zetterberg et al is unlikely to be from sample processing or assay differences. The same SiMoA technology was used for both studies. Previously, researchers have demonstrated that multiple freeze-thaw cycles [46] and delayed freezing [47] do not significantly influence serum Nf-L concentrations. Regardless, all blood samples in the present study were centrifuged immediately, whereafter the serum was aliquoted and frozen at -80°C. Assays were performed on serum that experienced minimal freeze-thaw cycles–i.e., no more than two.

In healthy control sheep, we observed a trend toward higher Nf-L concentrations in young lambs. This is consistent with studies on human neonatal development that show Nf-L levels increasing after birth for the first 12 postnatal weeks of life [48]. Further research is necessary to characterize the dynamics of post-natal serum Nf-L concentrations in non-human species.

At the end of this experiment, sheep were confirmed PrP^Sc^ positive by EIA. Lymphoid tissues of the sheep were not consistently positive. Low EIA values and non-detectable PrP^Sc^ in lymphoid tissue were unexpected findings in this study. Classical scrapie in sheep is lymphotropic and generally presents with abundant detectable PrP^Sc^ in lymphoid tissues, especially tonsils and retropharyngeal lymph nodes [49, 50]. The reason for inconsistent or lack of PrP^Sc^ accumulation in these sheep is unknown. The sheep *PRNP* genotype was VRQ/ARQ and therefore should be susceptible to scrapie via the oronasal route. A previous study using the same inoculation route and dose of classical in sheep with the VRQ/ARQ *PRNP* genotype showed differences in degree of lymphoid accumulation between x124 and 13–7 sheep at end stage clinical disease [37]. It’s likely that the combination of sheep genotype and classical scrapie strain x124 contributed to the paucity of lymphoid PrP^Sc^ accumulation in the present study. This will be the focus of future investigations.

The number of lymphoid follicles in rectal biopsies decreased as the study progressed. Animals tended to have higher follicle count early in the experiment compared to later time points. A large drop-off in follicle number occurs around 6 mpi corresponding to about 9 months of age. This finding was consistent with other studies. As animals age, the quantity of their mucosal lymphoid tissue declines [51, 52]. It’s interesting that 7/12 of RT-QuIC positive rectal biopsy tissues were from 6 mpi or earlier; however, the presence of lymphoid follicles in the rectal biopsies was not requisite for positive RT-QuIC based on lymphoid follicle counts compared to the RT-QuIC tissue results. These findings were similar to a study in elk with chronic wasting disease that suggested follicles may not be necessary for amplifying PrP^Sc^ from rectal mucosa tissue with RT-QuIC [44].

In conclusion, serum Nf-L did not identify early pre-symptomatic sheep that were experimentally inoculated with classical scrapie strain x124. Serum Nf-L could be used to easily identify sheep with neurologic deficits. This may be particularly useful for clinical presentations of scrapie that are intermittent during the initial period when neurologic signs are emerging. The present study was limited by a low number of animals examined, a single prion protein genotype, and one classical scrapie strain. Future studies should be performed in veterinary species including sheep to characterize Nf-L concentrations in other disease conditions. Various neurologic diseases such as meningitis or brain abscesses, for example, would be expected to elevate serum Nf-L; therefore, Nf-L should be regarded as a non-specific biomarker from a broad diagnostic perspective.

## Supporting information

S1 Table Timepoint serum neurofilament concentrations from sheep inoculated with the scrapie agent. (CSV)
